# Effective Manipulation of Spin Dynamics by Polarization Electric Field in InGaN/GaN Quantum Wells at Room Temperature

**DOI:** 10.1002/advs.201903400

**Published:** 2020-06-01

**Authors:** Xingchen Liu, Ning Tang, Shixiong Zhang, Xiaoyue Zhang, Hongming Guan, Yunfan Zhang, Xuan Qian, Yang Ji, Weikun Ge, Bo Shen

**Affiliations:** ^1^ State Key Laboratory of Artificial Microstructure and Mesoscopic Physics School of Physics Peking University Beijing 100871 China; ^2^ Frontiers Science Center for Nano‐optoelectronics & Collaborative Innovation Center of Quantum Matter Peking University Beijing 100871 China; ^3^ State Key Laboratory for Superlattices and Microstructures Institute of Semiconductors Chinese Academy of Sciences Beijing 100083 China; ^4^ College of Materials Science and Opto‐Electronic Technology College of Physical Sciences University of Chinese Academy of Sciences Beijing 100049 China

**Keywords:** III‐nitride semiconductors, spin dynamics, spin–orbit coupling, time‐resolved Kerr rotation

## Abstract

III‐nitride wide bandgap semiconductors are favorable materials for developing room temperature spintronic devices. The effective manipulation of spin dynamics is a critical request to realize spin field‐effect transistor (FET). In this work, the dependence of the spin relaxation time on external strain‐induced polarization electric field is investigated in InGaN/GaN multiple quantum wells (MQWs) by time‐resolved Kerr rotation spectroscopy. Owing to the almost canceled two different spin–orbit coupling (SOC), the spin relaxation time as long as 311 ps in the MQWs is obtained at room temperature, being much longer than that in bulk GaN. Furthermore, upon applying an external uniaxial strain, the spin relaxation time decreases sensitively, which originates from the breaking of the *SU*(2) symmetry. The extracted ratio of the SOC coefficients shows a linear dependence on the external strain, confirming the essential role of the polarization electric field. This effective manipulation of the spin relaxation time sheds light on GaN‐based nonballistic spin FET working at room temperature.

## Graphical abstract

GaN‐based wide bandgap semiconductors hold great potential in developing spintronic devices working at room temperature. The effective manipulation of the spin dynamics by strain‐induced polarization electric field is confirmed in InGaN/GaN quantum wells (QWs) using time resolved Kerr‐rotation spectroscopy. Such an effective manipulation paves a new approach toward using wurtzite nitride semiconductor multiple QWs structures in developing a nonballistic spin field‐effect transistor.

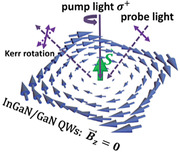

## 1 Introduction

Owing to their advantages of weak intrinsic spin–orbit coupling (SOC) and theoretically high Curie temperature of Mn‐doped GaN,^[^ ^1^ ^]^ III‐nitride wide bandgap semiconductors belong to the favorite materials in developing room temperature spintronic devices. Up to now, lateral nonlocal spin valves,^[^ ^2^ ^]^ spin light‐emitting diodes,^[^ ^3^ ^]^ and spin‐polarized diode lasers^[^ ^4^ ^]^ based on III‐nitride semiconductors have been implemented at room temperature. However, spin field‐effect transistor (FET) proposed by Datta and Das^[^ ^5^ ^]^ is still facing many challenges, such as the manipulation of the spin dynamics and maintenance of ballistic transport. The manipulation of the spin dynamics mainly depends on the effective magnetic field originating from the SOC. Generally, the SOC comes from either structural inversion asymmetry (SIA) or bulk inversion asymmetry (BIA). The SIA‐related SOC has attracted much concerns because it can be manipulated by an external electric field.^[^ ^6^ ^]^ While SOC induced by BIA could also be important due to its dependence on the crystal symmetry and can be tailored by artificial low‐dimensional quantum structures.^[^ ^7^ ^]^

The *SU*(2) symmetry broken induced by SOC terms results in nonconservative total spin operator $\vec{S}$ of electrons in BIA or SIA systems, i.e., $[\vec{S},H_{\mathrm{soc}}]\neq 0,$ where *H*_SOC_ refers to the SOC Hamiltonian. Mainly, the Dyakonov–Perel (DP) spin relaxation is regarded as the dominant mechanism in GaN‐based systems at room temperature.^[^ ^8^ ^]^ Under the framework of the DP mechanism, the spin relaxation mainly attributes to the wave vector $\vec{k}$‐dependent effective magnetic field (${\vec{\Omega }}_{\vec{k}}$). The components of ${\vec{\Omega }}_{\vec{k}}$, perpendicular to the spin, lead to the corresponding spin relaxation process. Interestingly, as a result of the total ${\vec{\Omega }}_{\vec{k}}$ being canceled out in DP mechanism dominant systems, the spin relaxation could be suppressed when identical but reversely signed Rashba and Dresselhaus SOC achieve. This phenomenon has been theoretically discussed in zinc blende and wurtzite‐based quantum wells (QWs).^[^ ^9^ ^]^ Some novel effects and devices have been predicted in semiconductor QWs with identical Rashba and Dresselhaus SOC terms, such as persistent spin helix (PSH)^[^ ^10^ ^]^ and nonballistic spin FET.^[^ ^11^ ^]^

## 2 Results

**Figure 1:**
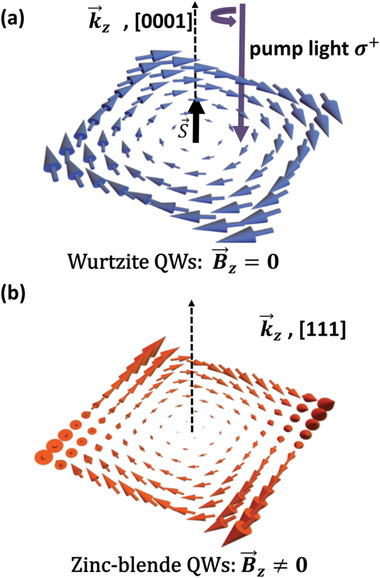
The schematic illustrations of the effective magnetic fields, and the cubic Dresshelhaus SOC term have been taken into consideration. The blue and red arrows represent the effective magnetic field. a) The spin polarization along *z* direction is induced by the circularly polarized pump beam in TRKR measurements. The out‐of‐plane component of effective magnetic fields is always zero in [0 0 0 1]‐grown wurtzite‐based QWs. b) The out‐of‐plane component of effective magnetic field is nonzero in [1 1 1]‐grown zinc blende‐based QWs, even if the identical Rashba and Dresselhaus SOC is achieved.

Up to now, an almost suppressed spin relaxation under external gate voltage has only been realized experimentally by Balocchi et al.^[^ ^12^ ^]^ in [1 1 1]‐grown zinc blende GaAs‐based QWs at low temperature. For a zinc‐blende system, the relaxation of all spin components could only be suppressed in [1 1 1]‐grown QWs. However, the effect of the cubic Dresselhaus terms becomes significant with increasing temperature. As a result, the out‐of‐plane component of ${\vec{\Omega }}_{\vec{k}}$ is nonzero and hence the relaxation process of the in‐plane spin could not be suppressed,^[^ ^11^, ^12^ ^]^ as shown in **Figure** **1**b. On the contrary, the out‐of‐plane component of ${\vec{\Omega }}_{\vec{k}}$ is always zero in [0 0 0 1]‐grown wurtzite GaN‐based QWs, as shown in Figure 1a, and the relaxation of all spin components could indeed be suppressed. Moreover, due to the controllable built‐in polarization electric field and strong quantum confinement caused by the large band offset, the suppressed spin relaxation without external manipulation could thus be achieved in wurtzite GaN‐based QWs. These characters of wurtzite GaN QWs make it possible to realize the “on” state of the nonballistic spin FET without constant voltage background. However, the manipulation of the spin dynamics, which is one of the key elements for implementing spin FET, is rarely investigated experimentally in wurtzite nitride semiconductors. In this work, the manipulation of the spin relaxation by external strain‐induced polarization field in InGaN/GaN multiple quantum wells (MQWs) was carefully investigated.

The SOC Hamiltonian in wurtzite InGaN/GaN [0 0 0 1]‐MQWs can be expressed as^[^ ^11^, ^13^ ^]^ $H_{\mathrm{soc}}=H_{\mathrm{soc}}^{\mathrm{SIA}}+H_{\mathrm{soc}}^{\mathrm{BIA}}=\frac{\hbar }{2}\;{\vec{\Omega }}_{\vec{k}}\cdot \vec{\sigma }$, and the effective magnetic field ${\vec{\Omega }}_{\vec{k}}$ has the following form:

(1) $${\vec{\Omega }}_{\vec{k}}=\frac{2}{\hbar }\left(\begin{array}{c} \left[\alpha_{Q}+\gamma^{1}-\gamma_{D}^{3}\left(k_{∥}^{2}-bk_{z}^{2}\right)\right]k_{y}\\ -\left[\alpha_{Q}+\gamma^{1}-\gamma_{D}^{3}\left(k_{∥}^{2}-bk_{z}^{2}\right)\right]k_{x}\\ 0 \end{array}\right)$$

where $\vec{k}$, $\vec{\sigma }$, and *b* respectively correspond to the electron momentum, Pauli matrices, and tight‐binding model parameter, which is equal to 3.959^[^ ^14^ ^]^ in wurtzite GaN. In Equation (1), the Dresselhaus SOC is described by $\gamma_{D}^{3}$, and the strength of the SOC induced by intrinsic wurtzite SIA and QWs’ SIA are given by *γ* ^1^ and *α*_Q_. Owing to the quantum confinement along *c*‐direction and low doping level in the system, i.e., $k_{z}^{2}\gg k_{∥}^{2}$, the cubic terms of the Dresselhaus SOC associated with $k_{∥}^{2}$ can be neglected.^[^ ^11^, ^12^ ^]^ Then, the effective magnetic field can be simplified as:

(2) $${\vec{\Omega }}_{\vec{k}}=\frac{2}{\hbar }\left(\begin{array}{c} \left[\alpha_{Q}+\gamma_{w}\right]k_{y}\\ -\left[\alpha_{Q}+\gamma_{w}\right]k_{x}\\ 0 \end{array}\right)$$

where the total SOC term related to the intrinsic wurtzite SIA and BIA is described by $\gamma_{w}=\gamma^{1}+\gamma_{D}^{3}bk_{z}^{2}$. Considering the quantum confinement and the polarization electric field in QWs, the $<k_{z}^{2}>$ can be estimated by $<k_{z}^{2}>={\left(\pi /d\right)}^{2}$, where *d* corresponds to the well width.^[^ ^12^, ^15^ ^]^ In one word, *γ*_w_ is mainly determined by the well width, while *α*_Q_ is attributed to the built‐in polarization electric field, which is affected by the In composition and could also be manipulated by external strain. In order to get nearly identical *α*_Q_ and *γ*_w_ and suppress the spin relaxation, an appropriate well width and In composition in the InGaN/GaN MQWs are designed. The structures of the InGaN/GaN MQWs used in our experiments are described in the Experimental Section.

**Figure 2:**
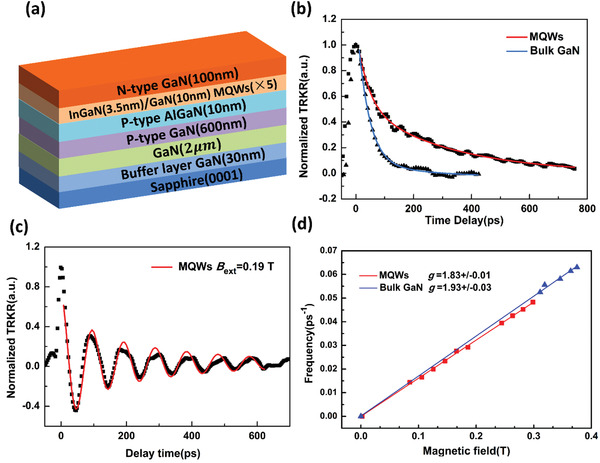
a) The schematic illustrations of sample A's structure. b) The TRKR results of InGaN/GaN MQWs and bulk GaN without external strain. c) The TRKR results of InGaN/GaN MQWs under an external magnetic field *B*_ext_ =  0.19 T. d) By fitting the frequency of Larmor precession under various magnetic fields, the extracted *g*‐factor of InGaN/GaN MQWs and bulk GaN are 1.83 ± 0.01 and 1.93 ± 0.03, respectively.

Time‐resolved Kerr rotation (TRKR) spectroscopy was adopted to measure the spin relaxation time of both the InGaN/GaN MQWs (sample A) and a bulk GaN (sample B, as reference) at room temperature (see the Experimental Section). First, the TRKR signals under various external magnetic fields were measured to extract the effective Landé *g*‐factor. As shown in **Figure** **2**d, the *g*‐factors of samples A and B were 1.83 and 1.93, clarifying that the TRKR signals originated from the InGaN/GaN MQWs in sample A^[^ ^16^ ^]^ and bulk GaN in sample B, respectively. Furthermore, the TRKR measurements without external magnetic field were conducted, as shown in Figure 2b. The extracted *τ*_s_ of bulk GaN and InGaN/GaN MQWs were 56 and 311 ps, respectively. It is evident that the spin relaxation time of InGaN/GaN MQWs is much longer than that of bulk GaN, indicating the almost suppressed spin relaxation in the MQWs. It is worth pointing out that the spin relaxation time of bulk GaN and QWs extracted from the TRKR signal with an external magnetic field (less than 0.4 T) is significantly longer than that in the zero magnetic field. The ratio of the spin relaxation time *τ*_s,B ≠ 0_/*τ*_s,B  =  0_ approaches 4/3. This result could be attributed to the anisotropy of ${\vec{\Omega }}_{\vec{k}}$ under the framework of the DP mechanism.^[^ ^8b^ ^]^ Due to the constraint of the equipment setup, the magnetic field and external strain could not be simultaneously applied. Based on that, we focused only on the spin relaxation time extracted from TRKR signal without external magnetic field.

## 3 Discussion

In order to fully understand the much longer spin relaxation time in MQWs, some theoretical analyses are performed. In the DP mechanism, the spin relaxation rate tensor can be expressed as^[^ ^8^, ^17^ ^]^ $\Gamma_{i,j}=\frac{1}{2}\left(\delta_{i,j}{\vec{\Omega }}_{\vec{k}}^{2}-\Omega_{i}\Omega_{j}\right)\tau_{p}$, where *τ*_p_ is the momentum scattering time. Combining the formula of ${\vec{\Omega }}_{\vec{k}}$, Γ_i,j_ can be calculated over the Boltzmann distributions at room temperature. As shown in Figure 1a, the electron spin induced by a circularly polarized pump beam is perpendicular to the *c*‐plane. Therefore, only the component Γ_z,z_ is considered and the spin relaxation rate related to the QWs’ SIA is

(3) $$\Gamma_{z,z}^{R}=4\alpha_{Q}^{2}m^{*}k_{B}T\tau_{p}/\hbar^{4}$$

while the intrinsic wurtzite SIA and BIA related relaxation rate is

(4) $$\Gamma_{z,z}^{W}=4\gamma_{W}^{2}m^{*}k_{B}T\tau_{p}/\hbar^{4}$$

There is an additional term in the spin relaxation rate which originates from the interference between QWs’ SIA term and intrinsic wurtzite SOC terms, and it can be written as

(5) $$\Gamma_{z,z}^{\mathrm{int}}=8\gamma_{W}\alpha_{Q}m^{*}k_{B}T\tau_{p}/\hbar^{4}$$

Taking all of the three terms into account, the total spin relaxation rate is $\Gamma_{z,z}=\Gamma_{z,z}^{R}+\Gamma_{z,z}^{W}+\Gamma_{z,z}^{\mathrm{int}}$ , and *τ*_s_ can then be expressed as

(6) $$\tau_{s}=1/\Gamma_{z,z}=\frac{\hbar^{4}}{4m^{*}k_{B}T\tau_{p}\gamma_{W}^{2}}\;\frac{1}{{\left(1+\alpha_{Q}/\gamma_{W}\right)}^{2}}$$

As indicated by Equation (6), the spin relaxation time will become infinite when the ratio $\frac{\alpha_{Q}}{\gamma_{W}}$ approaches −1, and the spin relaxation process is well suppressed. In contrast, owing to the absence of the interference term,^[^ ^8a^ ^]^ this suppression behavior is absent in bulk GaN. Given the much longer spin relaxation time extracted by TRKR, the DP spin relaxation might indeed be suppressed in InGaN/GaN MQWs. In order to confirm this assumption, the strain‐manipulated TRKR measurements are conducted.

It should be noted that the derivative of *τ*_s_ with respect to $\frac{\alpha_{Q}}{\gamma_{W}}$ $\left[\frac{d\tau_{s}}{d(\alpha_{Q}/\gamma_{W})}\right]$ also tends to be infinity when $\frac{\alpha_{Q}}{\gamma_{W}}=-1$ being satisfied, and the QWs’ SIA term could be manipulated by the external strain‐induced polarization field. As a result, the spin relaxation time should be extremely sensitive to the variation of the external strain upon this condition. The equipment used to apply external strain is described in Experimental Section. The additional polarization electric field induced by the external strain can be expressed as $E_{\mathrm{add}}=\left[e_{31}-e_{33}\left(\frac{c_{12}}{c_{13}}\right)\varepsilon_{\mathrm{yy}}\right]/\varepsilon_{\mathrm{GaN}}$, where *ε*_yy_ is the in‐plane external strain, *e*_31_ and *e*_33_ are the piezoelectric constants, *c*_12_ and *c*_13_ are the elastic constants, and *ε*_GaN_ is the dielectric constant.^[^ ^18^ ^]^ With the external strain increasing to *ε*_yy_ =  8.46  × 10^−4^ in the following experiments, the additional polarization field is estimated to be *E*_add_ =  2.58  × 10^5^ V  cm^−1^.

**Figure 3:**
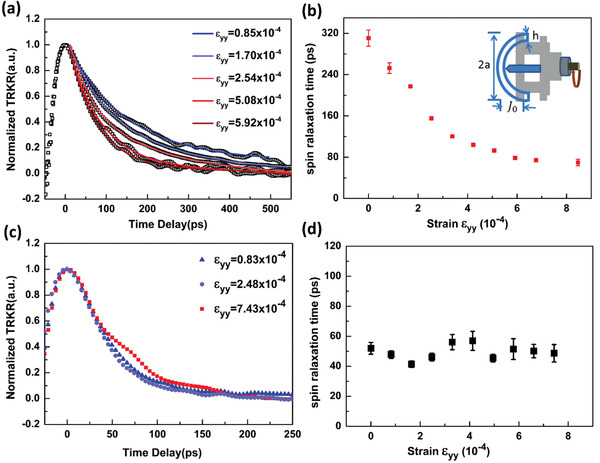
a) The TRKR signals of InGaN/GaN MQWs under external strains. b) The extracted *τ*_s_ of InGaN/GaN MQWs under various strains. The insets show the equipment used to apply external uniaxial strain. c) The TRKR signals of bulk GaN under external strains. d) The extracted *τ*_s_ of bulk GaN under various strains.

The spin relaxation time of MQWs and bulk GaN under various strains are shown in **Figure** **3**. The spin relaxation time of the InGaN/GaN MQWs decreases dramatically with increasing the external strain, while the spin relaxation time of the bulk GaN is nearly independent of the external strain. The sensitive dependence of the spin relaxation time on external strain and the initial long spin relaxation time in the InGaN/GaN MQWs all reveal the fact of the cancellation of the SOC terms. As discussed above, the absence of similar behavior in bulk GaN mainly attributes to the negligible interference term over there. In the meantime, the photoexcited carrier lifetime is nearly independent of the external strain in the MQWs, confirming that the variation of the overlap between electron and hole wave functions is negligible. In this situation, the suppressing of the interface scattering with increasing external strain should also be negligible, and the momentum scattering time could hence be regarded as constant. To directly confirm the cancellation of the SOC terms, the ratios $\frac{\alpha_{Q}}{\gamma_{W}}$ are extracted for different *τ*_s_ by using Equation (6) in the InGaN/GaN MQWs. Material parameters used in the calculations are given as follows. In the In_*x*_Ga_1 − *x*_N/GaN MQWs, the effective electron mass is m* =  (0.2 − 0.09*x*)*m*_0_^[^ ^13^ ^]^; the momentum scattering time is set to 40 fs;^[^ ^8a^ ^]^ and the intrinsic wurtzite SIA and BIA coefficients are *γ* ^1^ =  9 meVÅ^[^ ^19^ ^]^ and $\gamma_{D}^{3}=0.33\;\mathrm{eV}$Å^3^.^[^ ^14^ ^]^ Due to the lack of accurate coefficients for InGaN, the intrinsic SIA and BIA coefficients of GaN, which would not be deviated too far from that of InGaN with low In composition, were adopted in the calculation. In addition, the initial total polarization electric field in InGaN/GaN MQWs is estimated to be  2.08  × 10^6^ V cm^−1^.^[^ ^20^ ^]^

**Figure 4:**
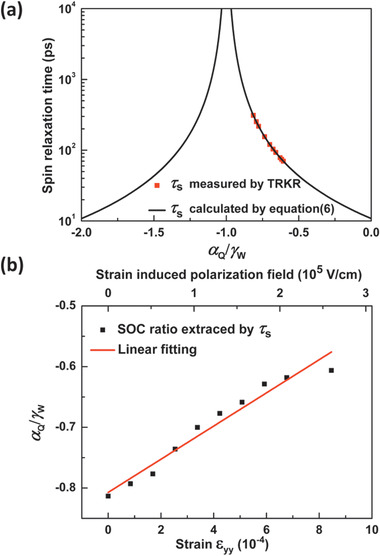
a) The dependence of *τ*_s_ on the ratios between the QW's SIA and intrinsic wurtzite SOC coefficients calculated by Equation (6) in InGaN/GaN MQWs. The red points represent *τ*_s_ measured by TRKR and corresponding *α*_Q_/*γ*_W_ under various strains. b) The extracted ratios *α*_Q_/*γ*_W_ vary as a function of the external strain and polarization electric field, and the red line shows the linear fitting.

As shown in **Figure** **4**a, the dependence of the spin relaxation time on $\frac{\alpha_{Q}}{\gamma_{W}}$ is calculated and exhibits as a curve, while the red points represent the *τ*_s_ measured by TRKR and corresponding $\frac{\alpha_{Q}}{\gamma_{W}}$ under various external strains. The extracted SOC ratio $\frac{\alpha_{Q}}{\gamma_{W}}$, initially approaching −0.81, is indeed close to the DP spin relaxation suppression condition. The initial polarization field is weakened by the external strain and the ratio *α*_Q_/*γ*_W_ increases from −0.81 to −0.61. Therefore, based on the current structures, the higher initial polarization field (associated with a higher In composition) and narrower well width are needed to realize the ideal ratio $\frac{\alpha_{Q}}{\gamma_{W}}=-1$. However, in those cases, the In alloy composition fluctuations (originating from higher In composition) and monolayer fluctuations (originating from narrower well width) would become more significant. The *SU*(2) symmetry ($\frac{\alpha_{Q}}{\gamma_{W}}=-1$) would be broken at the fluctuation point, and the longer spin relaxation time is limited. In order to overcome these fluctuations, a better growth method may work, such as molecular beam epitaxy.^[^ ^21^ ^]^ Moving forward, due to the Elliott–Yafet (EY) mechanism and hyperfine‐field‐induced spin relaxation,^[^ ^2b^ ^]^ the crystal quality should be improved to further prolong *τ*_s_ even if the ideal ratio $\frac{\alpha_{Q}}{\gamma_{W}}=-1$ has been realized.

The initial SOC coefficients induced by the QWs’ SIA is estimated to be *α*_Q_ =   − 15.81 meVÅ , which is consistent with the theoretical calculations in GaN and InN‐based QWs.^[^ ^9^, ^11^ ^]^ Notably, *α*_*Q*_ is extracted from *τ*_s_, and the spin relaxation is attributed to the DP mechanism in our calculations. Although it is a good approximation,^[^ ^8^ ^]^ there still exist some other relaxation channels such as EY mechanism and hyperfine field. As a result, the SOC coefficient *α*_*Q*_ is slightly overestimated. Furthermore, the extracted $\frac{\alpha_{Q}}{\gamma_{W}}$ shows a linear dependence on the external strain, as shown in Figure 4b. It has been demonstrated that the QWs’ SIA terms linearly depend on the external strain‐induced electric field,^[^ ^22^ ^]^ whereas the intrinsic wurtzite SOC terms are almost independent of strain in the range of less than 10^−3^ in our experiments.^[^ ^23^ ^]^ Eventually, the linear dependence of the polarization electric field on external strain results in the linear dependence of $\frac{\alpha_{Q}}{\gamma_{W}}$ on external strain in our experiments. More importantly, this linear dependence confirms that the manipulation of the spin dynamics mainly attributes to the strain‐induced polarization electric field, which is essentially practical for spin FET applications.

## 4 Conclusion

In conclusion, we have confirmed that the ratio of the SOC coefficients $\frac{\alpha_{Q}}{\gamma_{W}}$, initially equaling to −0.81, approaches to the spin relaxation suppression condition in the InGaN/GaN MQWs used in our experiments. As a result, the spin relaxation time in InGaN/GaN MQWs is much longer than that in bulk GaN and could be effectively manipulated by strain‐induced polarization field. The much longer spin relaxation time will surely benefit the spin transport process and greatly enhance the performance of relevant spintronic devices. In the meantime, the external uniaxial strain manipulates the spin dynamics mainly by internal polarization electric field; hence, it is basically an electric field manipulation, which is essentially practical. So, the effective manipulation of the spin relaxation time may advance the development of a nonballistic spin FET based on InGaN/GaN MQWs working at room temperature. While due to the negligible SOC interference term, these excellent properties are absent in bulk GaN. This work thus proposes a new approach toward using wurtzite nitride MQWs structures in developing spintronic devices.

## 5 Experimental Section

5.1

5.1.1

##### Sample Growth

Both the InGaN/GaN MQWs (sample A, shown in Figure 2a) and bulk GaN (sample B, reference sample) were grown by mental‐organic chemical vapor deposition (MOCVD). Sample A consisted of 2 µm undoped GaN layer grown on a *c*‐axis sapphire substrate with 30 nm undoped GaN buffer layer on top, followed by 600 nm p‐GaN (Mg‐doped: 1  × 10^18^ cm^−3^) and 10 nm p‐Al_0.18_GaN_0.82_N (Mg‐doped: 1  × 10^17^ cm^−3^), five periods of 3.5 nm undoped In_0.13_Ga_0.87_N wells and 10 nm undoped GaN barriers, and 100 nm n‐GaN (Si‐doped: 1  × 10^18^ cm^−3^). Specifically, the background carrier density in QWs was estimated to 1  × 10^17^ cm^−3^ at room temperature. The stack structures of sample B were 2 µm undoped GaN and 2.5 µm n‐GaN (Si‐doped: 1  × 10^18^ cm^−3^) grown on a *c*‐axis sapphire.

##### TRKR Measurements and Applying External Strain

In the TRKR measurements, the pump and probe beams were derived from the frequency‐double output of a femtosecond mode‐locked Ti:Sapphire laser, and the energy of the beam was 3.40 eV. The average power of the pump and probe beams approached to 10:1, and the estimated density of the photoexcited carriers was 1.17  × 10^15^ cm^−3^. A double exponential damped function $\left[A_{1}\exp\left(-t/\tau_{c}\right)+A_{2}\right]\exp\left(-\frac{t}{\tau_{s}}\right)$ was used to extract the spin relaxation time, where *τ*_c_ and *τ*_s_ corresponded to the photo‐generated carrier recombination time and spin relaxation time, respectively.^[^ ^8a^ ^]^

Applying an external magnetic field, which was parallel to the sample surface, would lead to Larmor precession of spin induced by the circular polarized pump beam, and the TRKR signals showed an oscillation behavior. The typical TRKR signal of sample A under an external magnetic field *B*_ext_ =  0.19 T was shown in Figure 1c. A double exponential cosine damped function [*A*_1_exp( − *t*/*τ*_c_) + *A*_2_]exp( − *t*/*τ*_s_)cos(*ωt*) was used to extract the spin relaxation time *τ*_s_ and oscillation frequency *ω*. In addition, *ω* could be expressed as *ω*  =  *g*μ_B_ *B*_ext_/ℏ, where *g* is the effective Landé *g*‐factor, μ_B_ is the Bohr magneton, and ℏ is the reduced Plank's constant. By fitting *ω* under various magnetic field *B*_ext_, the effective Landé *g*‐factors could be extracted.

The uniaxial strain equipment used in our experiments was composed of a micrometric system, as shown in the insets of Figure 3b. By increasing the displacement of the micrometric system, a tension strain along the long axis of the sample would occur. The in‐plane strain could simply be expressed as *ε*_yy_ =  3*hJ*_0_2*a* ^2^, where 2*a* is the length of the strip, *J*_0_ is the replacement of the micrometric, and *h* is the thickness of the sample.^[^ ^24^ ^]^

## Conflict of Interest

The authors declare no conflict of interest.
